# The number of optometrists is inversely correlated with blindness in OECD countries

**DOI:** 10.1111/opo.12746

**Published:** 2020-10-16

**Authors:** Einat Shneor, Michal Isaacson, Ariela Gordon‐Shaag

**Affiliations:** ^1^ Department of Optometry and Vision Science Hadassah Academic College Jerusalem Israel; ^2^ Department of Gerontology Faculty of Social Welfare & Health Sciences University of Haifa Haifa Israel

In the article “Estimated number of ophthalmologists worldwide (International Council of Ophthalmology update): will we meet the needs?” Resnikoff *et al*.^1^ surveyed the density (defined as the number per million people) of ophthalmologists (OMDs) globally using questionnaires sent to national ophthalmology councils or Ministries of Health. The questionnaire queried the number of OMDs in each country as well as the number of OMDs who routinely perform refraction. They tested whether there was a correlation between OMDs’ density and mild to severe vision impairment (MSVI) or blindness, using data collected elsewhere as part of a worldwide survey on these conditions.^2^ The paper reported a weak, inverse correlation between the prevalence of blindness and the OMDs’ density. However, they did not find a correlation between the density of OMDs performing refraction and MSVI. The authors suggest that this may be due to refraction being performed by optometrists and other allied eyecare workers in some countries, since these health providers were not included in the survey.

As a response to these findings, seeking what we understand as a missing link, we examined the density of OMDs together with optometrists in relation to blindness and MSVI. We hypothesise that the combined number of optometrists and OMDs will have a significant inverse correlation with blindness and MSVI. We think that this correlation exists since in developed countries, both professions are essential providers of eye care. We tested this notion on Organisation for Economic Cooperation and Development (OECD) countries (*n* = 37) since reliable and accessible data regarding the number of optometrists per population and their scope of practice exists for these countries. Furthermore, in most of these countries, optometrists are trained to practice at the World Council of Optometry (WCO) level 3, which includes investigation, examination and evaluation of the eye and adnexa to detect, diagnose and manage disease.^3^ We reasoned that when eye care practitioners detect pathology during routine refraction examinations, it will lead to a reduction in blindness.

Data on the density of optometrists was available for 34 of the OECD countries (*Table* *1*) via the following sources: the ECOO Bluebook^4^ (*n* = 24) or the WCO Bluebook^5^ (which was obtained in a personal communication from the WCO, *n* = 3, Chile, Colombia and Japan) unless a more recent source was available such as the national optometry council or government information (*n* = 6, Australia,^6^ Canada,^7^ Israel,^8^ Mexico,^9^ New Zealand^10^ and USA^11^), or from personal communication (*n* = 1, Iceland). For three of the 37 OECD countries (South Korea, Lithuania and Luxembourg) data on the number of optometrists was not available, so they were not included in this analysis.

**Table 1:** Data on the number of optometrists and the scope of practice in OECD countries

Country	WCO Optometry level	Optometrists Per Million Population	OMD^1^ and Optometrists per million	OMD who perform refraction^1^ and optometrists per million
Australia	4*	243.6^6^	283.7	248.6
Austria	3*	104.0^4^	214.6	173.1
Belgium	3	35.0^4^	132.4	NR
Canada	4*	176.9^7^	210.9	189.6
Chile	3*	2.0^5^	51.8	45.5
Colombia	4*	109.0^5^	140.1	136.2
Czech Republic	3	95.0^4^	195.5	132.7
Denmark	3	318.0^4^	397.4	387.5
Estonia	3	206.0^4^	305.0	218.4
Finland	3*	269.0^4^	350.8	340.5
France	2	47.0^4^	139.0	127.5
Germany	3	211.0^4^	301.5	290.2
Greece	2	183.0^4^	365.6	342.7
Hungary	2	71.0^4^	168.4	83.2
Iceland	3	121.6^†^	215.7	203.9
Ireland	3*	149.0^4^	187.4	163.4
Israel	3	314.4^8^	395.0	324.5
Italy	2	42.0^4^	159.1	144.4
Japan	NA	63.0^5^	177.1	77.3
Korea South (Republic of Korea)	NA	NA	NA	NA
Latvia	3	61.0^4^	188.9	NR
Lithuania	NA	NA	NA	NA
Luxembourg	NA	NA	NA	NA
Mexico	NA	59.0^9^	101.5	96.2
Netherlands	3*	70.0^4^	107.6	NR
New Zealand	4*	161.2^10^	189.0	164.7
Norway	3*	288.0^4^	369.0	358.9
Poland	2	44.0^4^	162.2	NR
Portugal	3	164.0^4^	222.0	214.7
Slovakia	3	48.0^4^	167.8	NR
Slovenia	2	12.0^4^	74.9	19.9
Spain	3	363.0^4^	468.5	428.9
Sweden	3*	205.0^4^	297.0	285.5
Switzerland	3*	120.0^4^	220.0	207.5
Turkey	1	0.0^4^	44.5	38.9
United Kingdom	4*	230.0^4^	276.4	235.8
United States	4*	122.5^11^	177.2	156.7

NA, Data was not available; NR, Data was not reported in Resnikoff *et al*.^1^; OMD, ophthalmologists; WCO, World Council of Optometry.

* Optometrists in these countries use at least diagnostic pharmaceuticals.

^†^ Personal communication.

For these 34 countries, data on the density of OMDs was available from Resnikoff *et al*.^1^ However, data on the density of OMDs performing refraction was only available for 29 of these countries.^1^ Data on the prevalence of blindness and MSVI^2^ were taken from Bourne *et al*.^12^ which is the same source as used in the Resnikoff study (*Table* *1*).

To test if the scope of optometry practice influences global blindness and MSVI, we categorized the scope of practice in each country according to the WCO competency‐based level of practice.^13^ For 2 of the 34 OECD countries (Mexico and Japan) data on the scope of practice was not available, so they were not included in this analysis. The WCO levels are characterised as follows and each level includes the level below it.^13^ Level 4 includes ocular therapeutic services. Level 3, ocular diagnostic services, including investigation, examination and evaluation of the eye and adnexa, and associated systemic factors to detect, diagnose and manage disease with or without pharmaceuticals. If optometry scope of practice included detection of ocular pathology and referral to physicians in the ECOO or WCO Blue Books,^4^, ^5^ it was defined as level 3. Level 2 is characterised by visual function service and level 1 is limited to optical technological services.

Correlations were analysed from data related to the density of OMDs and/or optometrists and continuous variables (prevalence rates of blindness or MSVI) using logistic regression in SPSS, version 25 (IBM, www.ibm.com). To compare with Resnikoff *et al*.^1^ we used the same criteria for correlations (*R* ^2^ < 0.09 were considered weak).

Resnikoff *et al*.^1^ found a weak correlation between the density of OMDs and blindness but not between OMDs doing refraction and MSVI (*R* ^2^ = 0.02, for both, although only the former is significant). In this study, no correlation was found between the density of OMDs (altogether or just those who do refraction) and blindness or MSVI (*Table* *2*). However, a significant inverse correlation was found between the density of optometrists and blindness (*R* = −0.40, *p* = 0.02), although no correlation was observed with MSVI (*R* = −0.24, *p* = 0.17). Furthermore, when the density of both optometrists and OMDs (altogether and those doing refraction) was taken into consideration, there was a significant inverse correlation both with blindness (*R* = −0.46, *p* = 0.007) and with MSVI (*R* = −0.34, *p* = 0.05).

**Table 2:** Linear regression between the number of ophthalmologists and optometrists and blindness or mild to severe vision impairment

	Blindness	MSVI
OMD (*N* = 34)	*p* = 0.18, *R* = −0.24, *R* ^2^ = 0.06	*p* = 0.05, *R* = −0.34, *R* ^2^ = 0.11
OMD doing refraction (*N* = 29)	*p* = 0.27, *R* = −0.21, *R* ^2^ = 0.05	*p* = 0.34, *R* = −0.18, *R* ^2^ = 0.03
Optometrists (*N* = 34)	***p* = 0.02, *R* = −0.40, *R*^2^ = 0.16**	*p* = 0.17, *R* = −0.24 *R* ^2^ = 0.06
OMD + Optometrists (*N* = 34)	***p* = 0.007, *R* = −0.46, *R*^2^ = 0.21**	***p* = 0.05, *R* = −0.34, *R*^2^ = 0.12**
OMD doing refraction + Optometrists (*N* = 29)	***p* = 0.009, *R* = −0.47, R^2^ = 0.22**	***p* = 0.05, *R* = −0.37, *R*^2^ = 0.14**
WCO level of Optometry (*N* = 32)	***p* = 0.03, *R* = −0.39, *R*^2^ = 0.16**	***p* = 0.05, *R* = −0.35, *R*^2^ = 0.12**
Resnikoff *et al*.^1^ all OMD	*R* ^2^ = 0.02	
Resnikoff *et al*.^1^ only OMD who perform refraction		*R* ^2^ = 0.02*

OMD, ophthalmologists; MSVI, mild to severe vision impairment.

* Not statistically significant.

The scope of practice is varied, but in 25 of the 32 countries, it was at least to WCO competency level 3 (*Table* *1*). In 19 of these 25 countries, optometrists detect and refer ocular disease and in another 6 they also manage disease with therapeutic pharmaceuticals (*Table* *1*). A significant inverse correlation was found between scope of optometry practice and MSVI (*R* = −0.35, *R* ^2^ = 0.12, *p* = 0.049) and blindness (*R* = −0.39, *R* ^2^ = 0.16, *p* = 0.03), as shown in *Table* *2*.

These results suggest that having more eye care practitioners lowers levels of blindness, even if the role for optometry in most of the countries listed is only detection and referral of ocular pathology (WCO level 3). Unfortunately, many people do not comply with the need for routine ocular health examinations. For example, only 63% of people with diabetes obtain the recommended annual fundus examination.^14^ However, many of these people present to their optometrist to get new spectacles. If optometrists use this opportunity for detection of ocular pathology and referral, it may lead to improved public eye health and reduction of blindness. This notion is supported by many studies that found that optometrists successfully diagnose and refer ocular pathology, for example see Gunn *et al*.^15^

Interestingly, the higher the scope of optometry practice, the lower the prevalence of MSVI and blindness. This further supports the notion that incidental findings in primary eye examinations may lead to better ocular health.

This study looked at the density of eye care practitioners. A different metric presented in the ECOO Bluebook^4^ is the percentage of refraction/primary care eye examinations carried out by OMDs. In 16 of the 28 countries for which they provide information, optometrists performed at least 50% of primary care examinations. Interestingly, some of the countries in which OMDs perform over 90% of the examinations have relatively few OMDs per population performing refraction. For example, Slovenia has 7.9 OMDs performing refraction per million population yet they perform over 90% of the refractions. In comparison, the UK has 5.8 OMDs performing refraction per million population and yet they provide less than 25% of the refractions. Thus, the percentage of OMDs performing refraction is not necessarily related to their density. In light of this, the inverse correlation between the total number of eye care practitioners and blindness is understandable.

The findings of this analysis highlight the importance of optometry in vision care as a cost‐effective way to improve vision health outcomes. While we examined data from OECD countries, considering our findings in a global context, lower income countries may benefit from the cost effectiveness and the higher accessibility rates associated with including optometry in vision care.

While the *R* ^2^ values in this study are not high, they are considerably higher than those found by Resnikoff^1^ using the same methodology. The purpose of this letter was to respond to and add to those findings regarding the role of optometry in the prevention of blindness and MSVI. While the results suggest that the density of optometrists has a positive impact on blindness and MSVI, we may be over‐estimating the relevance of the association, since the *R* ^2^ is low, while still statistically significant.

Another limitation of the analysis is in the correlative nature of the findings and the inability to establish causation. We did not control for any additional variables such as wealth. Further analysis may reveal that both the number of OMDs and optometrists correlate with wealth, which is a control variable known to be associated with good medical outcomes. However, all the countries in this analysis aside from two (Colombia and Turkey, who were upper middle income) were considered high income by the World Bank in terms of gross domestic product per capita.^16^ Thus, it is unlikely that wealth is the only factor that influenced blindness and MSVI.

## Disclosures

The authors report no conflicts of interest and have no proprietary interest in any of the materials mentioned in this article.

## Author contribution

**Einat Shneor:** Conceptualization (equal); Data curation (equal); Formal analysis (equal); Investigation (equal); Methodology (equal); Software (equal); Writing‐original draft (equal); Writing‐review & editing (equal). **Michal Isaacson:** Formal analysis (supporting); Methodology (supporting); Software (equal); Writing‐review & editing (supporting). **Ariela Gordon‐Shaag:** Conceptualization (equal); Data curation (equal); Formal analysis (equal); Methodology (equal); Software (equal); Writing‐original draft (equal); Writing‐review & editing (equal).
